# The Tip of the Iceberg: On the Roles of Regulatory Small RNAs in the Virulence of Enterohemorrhagic and Enteropathogenic *Escherichia coli*

**DOI:** 10.3389/fcimb.2016.00105

**Published:** 2016-09-21

**Authors:** Shantanu Bhatt, Marisa Egan, Valerie Jenkins, Sarah Muche, Jihad El-Fenej

**Affiliations:** Department of Biology, Saint Joseph's UniversityPhiladelphia, PA, USA

**Keywords:** transcriptional, posttranscriptional, sRNA, LEE, EHEC, EPEC

## Abstract

Enterohemorrhagic and enteropathogenic *Escherichia coli* are gastrointestinal pathogens that disrupt the intestinal microvilli to form attaching and effacing (A/E) lesions on infected cells and cause diarrhea. This pathomorphological trait is encoded within the pathogenicity island locus of enterocyte effacement (LEE). The LEE houses a type 3 secretion system (T3SS), which upon assembly bridges the bacterial cytosol to that of the host and enables the bacterium to traffic dozens of effectors into the host where they hijack regulatory and signal transduction pathways and contribute to bacterial colonization and disease. Owing to the importance of the LEE to EHEC and EPEC pathogenesis, much of the research on these pathogens has centered on its regulation. To date, over 40 proteinaceous factors have been identified that control the LEE at various hierarchical levels of gene expression. In contrast, RNA-based regulatory mechanisms that converge on the LEE have only just begun to be unraveled. In this minireview, we highlight major breakthroughs in small RNAs (sRNAs)-dependent regulation of the LEE, with an emphasis on their mechanisms of action and/or LEE-encoded targets.

## Epidemiology and pathogenesis of EHEC and EPEC

Enterohemorrhagic *Escherichia coli* (EHEC) and enteropathogenic *E. coli* (EPEC) belong to the attaching and effacing (A/E) family of pathogens that are major public health concerns worldwide (Mellies et al., 2007; Bhatt et al., 2011). During infection, A/E pathogens adhere intimately to host cells (attachment) and destroy cellular microvilli (effacement) to form A/E lesions. These ultrastructural changes limit the absorptive capacity of the intestinal cells, leading to diarrhea (Mellies et al., 2007; Bhatt et al., 2011). The ability of EHEC and EPEC to form A/E lesions is mediated by factors encoded within the pathogenicity island locus of enterocyte effacement (LEE; Mellies et al., 2007; Bhatt et al., 2011). The LEE encodes a type 3-secretion system (T3SS) that assembles in the bacterial extracytoplasmic space and matures to puncture the host cell membrane to directly connect the bacterial cytosol to that of the infected host (Mellies et al., 2007; Bhatt et al., 2011). Subsequently, the bacterium traffics diverse effector molecules into the infected host where they hijack host signal transduction pathways to aid bacterial colonization and cause disease (Kenny et al., 1997; Mellies et al., 2007; Croxen and Finlay, 2010; Bhatt et al., 2011). The essentiality of the LEE to EPEC and EHEC virulence has made it a focal point for regulatory studies. Over 40 proteinaceous factors, operating at every conceivable level of gene expression, have been identified. Structural and mechanistic studies have been performed on many of these (Bustamante et al., 2001; Haack et al., 2003; Mellies et al., 2007; Jimenez et al., 2010). By contrast, the roles of regulatory small RNAs (sRNAs) in the pathogenesis of A/E bacteria remain undercharacterized. The current minireview focuses on sRNAs implicated in EHEC and EPEC virulence, with an emphasis on their mode of action, regulated targets, and significance to pathophysiology.

## Structure, function, and advantages of sRNAs

sRNAs are heterogeneous molecules that range from ~50 to 500 nucleotides (Waters and Storz, 2009). The majority of sRNAs base-pair to target mRNAs and affect transcriptional elongation, mRNA stability, and/or translation (Waters and Storz, 2009; Papenfort and Vogel, 2010). Base-pairing sRNAs can be further classified as *cis*-encoded or *trans*-encoded on the basis of their site of synthesis with respect to the target(s) controlled by them. *Cis*-encoded sRNAs are specified at the same genetic locus as their target genes but from the complementary strand (Waters and Storz, 2009; Papenfort and Vogel, 2010). As such, *cis*-encoded sRNAs possess expansive tracts of perfect complementarity to their target mRNA. By contrast, *trans*-encoded sRNAs are synthesized from genomic loci that are located distantly from their target genes (Waters and Storz, 2009; Papenfort and Vogel, 2010). Consequently, they elicit their regulatory effects via shorter and discontinuous tracts of complementarity, often ranging between 6 and 25 base-pairs in length. Owing to their limited potential for heteroduplex formation, most *trans*-encoded, but not *cis*-encoded, sRNAs require an RNA chaperone to facilitate base-pairing with their partner. The most frequently employed bacterial RNA chaperone is the posttranscriptional factor Hfq (Waters and Storz, 2009; Papenfort and Vogel, 2010). Together, Hfq and Hfq-dependent sRNAs coregulate numerous biological processes including oxidative stress, acid stress, motility, quorum sensing, antibiotic resistance, and virulence, among others (Waters and Storz, 2009; Chao and Vogel, 2010; Papenfort and Vogel, 2010).

sRNAs bestow numerous advantages that enhance the regulatory and phenotypic range of their bacterial host. For instance, sRNAs are metabolically inexpensive and rapidly synthesized because they are small in size and forgo translation (Waters and Storz, 2009). Moreover, because most sRNAs function posttranscriptionally the response time for target gene expression is significantly reduced (Shimoni et al., 2007; Mehta et al., 2008; Waters and Storz, 2009; Beisel and Storz, 2010). Other advantages of sRNAs include the presence of multiple sequentially diverse base-pairing regions, flexible positioning of complementary base-pairing sites on their target mRNAs, ability to uncouple and differentially regulate polycistronic genes, and lower basal level of gene expression by facilitating message degradation (Perez and Groisman, 2009; Beisel and Storz, 2010, 2011; Durand and Storz, 2010; Papenfort and Vanderpool, 2015). These regulatory and mechanistic properties of sRNAs significantly expand the responsiveness of bacterial gene expression to a multitude of environmental cues. Thus, due to the numerous benefits afforded by sRNAs, it comes as no surprise that pathogens have readily assimilated riboregulatory mechanisms into virulence-associated pathways.

## sRNA-dependent regulation of the LEE

### Role of Hfq in regulation of the LEE

**Figure 1:**
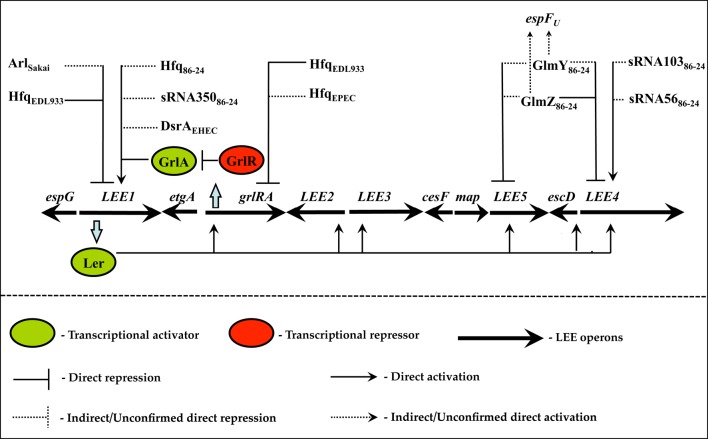
**Hfq and sRNA-dependent regulation of the LEE in EHEC and EPEC**. The locus of enterocyte effacement (LEE) pathogenicity island includes the multicistronic operons *LEE1-5*, the bicistronic operon *grlRA*, and multiple monocistronic transcription units. In an inducible environment the master regulator Ler orchestrates the synchronous transcriptional activation from the other LEE operons, including *grlRA*, which culminates with morphogenesis of A/E lesions. GrlA and GrlR participate in a complex positive and negative feedback loop with *ler* respectively to refine transcription from the LEE. In the EHEC strain EDL933 Hfq represses the LEE by destabilizing the *grlRA* mRNA as well as by targeting the 5′ UTR of *ler*. In EHEC 86-24 Hfq activates LEE via *ler*, and multiple trans-encoded sRNAs, integrated at different regulatory checkpoints, are involved in this regulation. These include sRNA350, sRNA103, sRNA56, GlmZ, and GlmY. In the EHEC strain Sakai the cis-encoded sRNA Arl silences LEE by repressing *ler*. In EPEC, Hfq represses the LEE by targeting grlRA. The figure has been modified from Bhatt et al. (2011).

Hfq is an RNA chaperone that functions as a homohexameric toroidal protein with distinct proximal and distal surfaces that facilitate sRNA-mRNA transactions (De Lay et al., 2013). Structural studies with Hfq reveal that its proximal surface binds to polyuridine tracts located downstream of a stem-loop—a structural feature abundant in Hfq-dependent sRNAs (Valentin-Hansen et al., 2004; De Lay et al., 2013). Meanwhile, the distal surface binds to tandem poly-(A-R-N) repeats, where A, R, and N represent adenine, purine, and any nucleotide respectively. *E. coli* mRNAs are replete with ARN repeats suggesting that Hfq preferentially associates with mRNAs by using its distal face (Link et al., 2009; De Lay et al., 2013). Hfq can simultaneously utilize its proximal and distal faces and facilitate sRNA-mRNA pairing (Link et al., 2009; De Lay et al., 2013). The relatively relaxed sequence recognition enables Hfq to control numerous cellular processes including virulence (Chao and Vogel, 2010). In both EHEC and EPEC Hfq controls the LEE with varying regulatory outcomes in a pathotype-specific manner (Hansen and Kaper, 2009; Shakhnovich et al., 2009; Kendall et al., 2011; Figure 1). In the EHEC strain EDL933, Hfq globally silences gene expression from the LEE through two independent regulatory pathways (Hansen and Kaper, 2009; Shakhnovich et al., 2009; Figure 1). In the exponential phase, inactivation of *hfq* stabilizes the *grlRA* mRNA (Hansen and Kaper, 2009). Because increased expression of *grlA* is epistatic to *grlR* this results in transcriptional activation of *ler*, which, in turn, activates the other LEE-encoded operons and stimulates pedestal formation in the *hfq* mutant. Meanwhile, in the stationary phase, the effect of Hfq is independent of *grlRA* since Hfq-dependent repression of the LEE is intact in the *grlRA* mutant (Hansen and Kaper, 2009). Presumably, this effect involves direct translational repression of *ler* since a *ler'-‘lacZ* translational fusion containing the 5′ UTR of *ler* that is transcribed from a heterologous GrlA-independent promoter is still regulatable by Hfq (Shakhnovich et al., 2009; Figure 1). Curiously, in the related EHEC biotype 86-24, the *hfq* mutant exhibits a starkly contrasting phenotype compared to the *hfq* mutant of EHEC EDL933 (Kendall et al., 2011). In EHEC 86-24, loss of *hfq* globally diminishes gene expression from the LEE in a *ler*-dependent manner. This suggests that Hfq functions as an activator, rather than a repressor, of the LEE in EHEC 86-24 (Kendall et al., 2011; Figure 1). However, whether the effect is direct or indirect remains to be elucidated. The antagonistic role of Hfq in EDL933 and 86-24 has been attributed to other genotypic differences such as the presence/absence of strain-specific sRNAs that lead to the observed regulatory outcomes. Whereas, the physiological role of Hfq in EHEC virulence has received considerable attention, the role of Hfq in EPEC has only been investigated superficially. In EPEC, inactivation of *hfq* derepresses the expression of GrlA and the GrlA-regulated LEE genes, suggesting that Hfq has a similar role to that observed in the EHEC strain EDL933 (Hansen and Kaper, 2009; Shakhnovich et al., 2009; Figure 1). However, the molecular details in EPEC have not been addressed.

### Role of Hfq-dependent trans-encoded sRNAs in regulation of the LEE

Ongoing studies have finally illuminated the elusive Hfq-dependent sRNAs that coregulate the LEE in EHEC 86-24. Using RNA sequencing, Gruber and Sperandio identified seven novel EHEC-specific sRNAs (Gruber and Sperandio, 2015). The expression of all but one of these sRNAs was diminished in the *hfq* mutant. Multiple Hfq-dependent sRNAs—sRNA350, sRNA103, and sRNA56—were shown to activate the LEE by affecting different targets and to varying degrees (Gruber and Sperandio, 2015; Figure 1). For instance, overexpression of sRNA350 globally activated transcription from all the LEE-encoded operons by affecting the master regulator *ler* (Gruber and Sperandio, 2015; Figure 1). However, the direct target of this riboregulator remains to be determined. Interestingly, the genetic architecture of sRNA350 does not conform to that of prototypical Hfq-dependent sRNAs. Most Hfq-dependent sRNAs are encoded by monocistronic transcription units; however, sRNA350 is encoded by the 3′ UTR of the LEE-encoded *cesF* gene, which specifies the chaperone for the T3S effector protein EspF (Elliott et al., 2002). Furthermore, sRNA350 does not appear to be posttranscriptionally cleaved and exerts its regulatory effect as part of the *cesF* transcript (Gruber and Sperandio, 2015). In contrast to sRNA350, sRNA103 and sRNA56 selectively target the *LEE4*-encoded *espA* transcript with the former eliciting a stronger regulatory response. However, neither sRNA appears to be complementary to *espA*, suggesting that the observed regulatory effect is mediated indirectly via an intermediate factor (Gruber and Sperandio, 2015). sRNA103 and sRNA56 also affect other genes scattered elsewhere in the EHEC genome (Gruber and Sperandio, 2015).

Besides EHEC-specific sRNAs, ancestral sRNAs, conserved between non-pathogenic and pathogenic lineages of *E. coli*, also regulate the LEE in EHEC 86-24. The two conserved Hfq-dependent sRNAs—GlmY and GlmZ—control the expression from the *LEE4* and *LEE5* operons as well as the non-LEE encoded gene *espF*_*U*_ (Gruber and Sperandio, 2014, 2015). GlmY and GlmZ are paralogous sRNAs that were originally identified as translational activators of the enzyme Glucoseamine-6-phosphate synthase (GlmS; Kalamorz et al., 2007; Reichenbach et al., 2008; Urban and Vogel, 2008; Waters and Storz, 2009). Despite extensive identity, GlmY and GlmZ exert their regulatory effects via distinct mechanisms. Unprocessed GlmZ possesses a seed region that base-pairs to and activates translation from the *glmS* transcript. GlmY, however, lacks the seed region and therefore does not base-pair to *glmS*. Rather, GlmY functions indirectly by preventing the processing of GlmZ by the enzyme RapZ, thereby increasing the cellular availability of unprocessed GlmZ to promote translation from *glmS* (Kalamorz et al., 2007; Reichenbach et al., 2008; Urban and Vogel, 2008; Waters and Storz, 2009). In EHEC, both GlmY and GlmZ destabilize the *LEE4* and *LEE5* encoded polycistronic transcripts while enhancing translation of *espF*_*U*_ by promoting cleavage in the intergenic region of the *espJ-espF*_*U*_ transcript (Gruber and Sperandio, 2014; Figure 1). GlmZ directly base-pairs to the *LEE4* transcript and selectively destabilizes the 3′ segment, containing *espADB* and the downstream ORFs, while having no effect on the 5′ segment of the transcript that contains *sepL* (Gruber and Sperandio, 2014). Direct evidence for duplex formation between GlmZ and *LEE4* was provided by site-directed mutations within the seed region of GlmZ as well as compensatory mutations in its target site on *LEE4*. Furthermore, in a subsequent study the same authors clarified the role of GlmY in regulation of the *LEE4* operon (Gruber and Sperandio, 2015). Here, they demonstrated that increased gene expression from the LEE and the ensuing A/E lesion formation observed in the Δ*glmY* mutant is abolished in the Δ*glmY* Δ*rapZ* double mutant suggesting that *rapZ* is epistatic (or downstream) to *glmY* (Gruber and Sperandio, 2015). This observation, coupled to the fact that overexpression of GlmY represses the *LEE4* transcript without affecting the *LEE4* promoter activity, suggest that GlmY post transcriptionally represses *LEE4* indirectly by binding to and sequestering RapZ from GlmZ. Free GlmZ, in turn, directly binds to and destabilizes the *LEE4* transcript thereby reducing pedestal formation. In other words, the GlmY- and GlmZ- dependent regulation of *LEE4* occurs in a manner similar to how these paralogs affect the expression of *glmS*. Interestingly, EspA, EspD, EspB, and some of the other downstream-encoded proteins are structural components of the T3S translocon, whereas SepL, along with SepD, functions as a regulatory switch that promotes the hierarchical secretion of translocators over effectors (Wang et al., 2008). SepL binds to effectors, such as Tir, effectively sequestering them until the maturation of the T3SS (Wang et al., 2008). Thereafter, the SepL/SepD switch triggers the shift from translocator to effector secretion. Perhaps, GlmZ and GlmY are expressed after the assembly of the T3SS when EspA, EspB, and EspD are no longer required but SepL is still needed to synchronize the hierarchical order of effector secretion, including EspF_*U*_. Paradoxically, a counterintuitive discovery made in this study was that GlmY and GlmZ antagonistically regulated targets all of which are required for the morphogenesis of pedestals in EHEC (Gruber and Sperandio, 2014). For instance, the proteinaceous factors encoded within *LEE4, LEE5*, and *espF*_*U*_ promote A/E lesions in EHEC; however, GlmY and GlmZ negatively regulated *LEE4* and *LEE5* but positively regulated *espF*_*U*_. The authors propose an attractive hypothesis that perhaps these regulatory sRNAs limit the uncontrolled expression from the LEE and synchronize it with the non-LEE encoded gene *espF*_*U*_ (Gruber and Sperandio, 2014). This mechanism would ensure that physiologically precise stoichiometric ratios of the architectural and secreted proteins are synthesized, which has been shown to be critical for the formation of A/E lesions and successful infection of the host. However, it remains to be determined if the sRNA-dependent regulation observed in EHEC 86-24 extends to other biotypes of EHEC, which form pedestals by the same mechanism. Interestingly, the linker protein EspF_U_, essential for EHEC to form pedestals, is not present in the genome of EPEC and the bacterium relies on a different posttranslational mechanism for pedestal formation (Mellies et al., 2007). This observation suggests that GlmY and GlmZ are unlikely to be functionally equivalent in EHEC and EPEC with regards to pedestal formation. This regulatory divergence between EHEC and EPEC appears to extend to another conserved Hfq-dependent sRNA—DsrA. In all tested strains of EHEC, DsrA activates the transcription of *ler* in an RpoS-dependent manner. However, DsrA does not affect the LEE in EPEC (Laaberki et al., 2006). This is in stark contrast to conserved proteinaceous transcription factors that regulate the LEE identically between EHEC and EPEC. For instance, the DNA-binding proteins H-NS, Fis, and GrlA modulate the LEE similarly in all A/E pathogens (Mellies et al., 2007; Bhatt et al., 2011). Thus, conserved sRNAs appear to be more malleable to regulatory rewiring in order to elicit strain-specific plastic responses for conserved morphogenetic pathways—a trait that is particularly advantageous in adapting pathogens to different niches. Moreover, these findings also suggest that it would be ill-advised to extrapolate the role of conserved sRNAs in one A/E pathogen based upon its role in another, and that their functions must be experimentally deduced in each member.

### Regulation of the LEE by *cis*-encoded sRNAs

Besides *trans*-encoded sRNAs, at least one *cis*-encoded sRNA, antisense regulator of *ler* RNA (*arl*), has been implicated in regulation of the LEE in the EHEC strain Sakai (Tobe et al., 2014; Figure 1). The *arl* gene is located downstream of *ler* but transcribed from the antisense strand. Consequently, Arl exhibits extensive complementarity to the *LEE1*-encoded *ler* mRNA. The transcription of *arl* is stimulated by elevated cytoplasmic levels of iron (Fe^2+^) or hydroxyl (OH⋅) radical but does not require the iron-responsive transcriptional factor Ferric uptake regulator (Fur; Tobe et al., 2014). Arl regulates the *ler*-encoded *LEE1* mRNA posttranscriptionally by specifically targeting the 3′ region of *ler*, over a region spanning the C-terminal domain of *ler* as well as the 3′ UTR. This conclusion is based on the observation that Arl-dependent regulation of *ler* is intact when just the C-terminal coding region of *ler* and its 3′ UTR is translationally fused to MBP and this chimeric *MBP'-‘ler* construct is transcriptionally driven by the heterologous *lac* promoter (Tobe et al., 2014). Moreover, Arl not only destabilizes the *LEE1* mRNA but also directly impacts translation completion from the *ler* ORF. As cytoplasmic iron is depleted the transcription of *arl* is reduced and this enhances the stability and translation from the *ler*-encoding *LEE1* mRNA, which in turn primes the LEE regulatory cascade that culminates with morphogenesis of A/E lesions (Tobe et al., 2014). These regulatory and phenotypic observations indisputably suggest that Arl posttranscriptionally controls the *LEE1* mRNA, presumably by direct base-pairing. However, the role of Arl in EPEC as well as the other EHEC biotypes has not been explored.

## Roles of sRNAs in other virulence-associated processes

In the past few years a novel class of sRNAs have been identified that specifically target other sRNA molecules (Tree et al., 2014). These sRNAs have been aptly termed “anti-sRNAs” since they mimic mRNA substrates and base-pair to complementary sRNAs to antagonize them. sRNA-anti-sRNA pairing may sequester and/or promote non-conducive conformations in the sRNA thereby preventing sRNA-mRNA base-pairing (Tree et al., 2014). The Hfq-dependent anti-sRNAs AgvB1 and AgvB2, encoded within an EHEC-specific prophage, confer a competitive advantage and enable the pathogen to colonize and multiply within the terminal rectal mucus (TRM) of the bovine gastrointestinal tract, thereby facilitating bacterial transmission and virulence (Tree et al., 2014). AgvB1 and AgvB2 mechanistically function by base-pairing to the core sRNA GcvB and antagonizing its effect. These anti-sRNAs possess the canonical base-pairing element, CACAACA, which is commonly observed on GcvB-regulated mRNAs and is recognized by the R1 seed region of GcvB (Sharma et al., 2011; Tree et al., 2014). Thus, AgvB1 and AgvB2 competitively inhibit GcvB by binding to it and sequestering the sRNA from its mRNA targets. The proposed mechanistic role of the AgvB paralogs is well supported by elegant genetic and biochemical experiments (Tree et al., 2014). However, the GcvB-dependent targets that affect colonization and transmission in the TRM are currently unknown. Another anti-sRNA, AsxR, is also encoded by an EHEC-specific prophage, BP933W. AsxR is also an Hfq-dependent anti-sRNA that duplexes with the sRNA FnrS and destabilizes it (Tree et al., 2014). However, the physiological role of AsxR in EHEC pathogenesis has not been addressed.

## Insights into RNA-mediated regulation and outstanding questions

It is evident that our understanding of the roles of sRNAs in the virulence of A/E pathogens is still in its infancy. By contrast, there is copious information on the roles of sRNAs in non-pathogenic *E. coli* and pathogenic *Salmonella* Typhimurium (Waters and Storz, 2009; Papenfort and Vogel, 2010). Even amongst A/E pathogens there is disproportionate investigation into the roles of sRNA. Whereas, dozens of novel sRNAs have been identified in EHEC, by contrast, sRNAs in EPEC remain cryptic (Gruber and Sperandio, 2014, 2015; Tree et al., 2014). Thus, there is dire need to explore the role of sRNAs in EPEC.

Perhaps the most significant question pertains to the number, nature, and location of riboregulatory genes in A/E pathogens. Multiple studies suggest that pathogenicity islands have a higher density of sRNA-coding genes (39 sRNAs/Mb) compared to the core genome (23 sRNAs/Mb; Raghavan et al., 2011; Keseler et al., 2013). Consistent with these observations, recently Tree et al. identified 63 novel Hfq-dependent sRNAs in EHEC, of which 55 were encoded within bacteriophage-derived pathogenicity islands and 8 within the core genome (Tree et al., 2014). These observations suggest A/E pathogens may possess a larger repertoire of sRNAs compared to their non-pathogenic siblings and may integrate more sRNAs per target. Horizontally acquired pathogenicity islands possess an unusually high AT-content, which is much higher than that of the core genome. This signature enables them to be readily assimilated into preexisting regulatory circuits to ensure that their expression is physiologically and evolutionarily tolerable and spatiotemporally coordinated with other genes (Fang and Rimsky, 2008; Perez and Groisman, 2009). It remains to be determined whether this selective pressure imposes constraints on the nucleotide composition of sRNAs encoded within pathogenicity islands, which in turn would be expected to affect their regulons. Other mechanisms for the evolution of sRNAs and their cognate targets have also been noted (Updegrove et al., 2015). It would be interesting to compare and contrast horizontally acquired sRNAs with ancestral sRNAs to determine the preferred mechanism(s) that influence their evolution and that of their targets. Conversely, a comparison of the regulons of orthologous sRNA between EHEC, EPEC, and *E. coli* would reveal patterns of gene acquisitions and losses between related bacterial strains. Such a study would be useful in further refining the principles that dictate the structural, functional, and mechanistic evolution of ancestral sRNAs.

In summary, our current knowledge on the roles of sRNAs in the virulence of A/E pathogens merely represents the tip of the iceberg. However, the implementation of genome-wide transcriptomic screens in EHEC and EPEC to rapidly identify virulence-associated sRNAs promises to usher in an era of explosive research, which will undoubtedly rival that of protein-based regulators.

## Author contributions

SB wrote the major body of the manuscript. ME, VJ, SM, and JE made equal contributions to the manuscript.

### Conflict of interest statement

The authors declare that the research was conducted in the absence of any commercial or financial relationships that could be construed as a potential conflict of interest.
